# Limited content overlap between commonly used self-report instruments for central (pain) sensitization in rheumatology

**DOI:** 10.1093/rap/rkae108

**Published:** 2024-08-26

**Authors:** Peter M ten Klooster, Jorge P Simoes, Harald E Vonkeman

**Affiliations:** Department of Psychology, Health and Technology, University of Twente, Enschede, The Netherlands; Department of Psychology, Health and Technology, University of Twente, Enschede, The Netherlands; Department of Psychology, Health and Technology, University of Twente, Enschede, The Netherlands; Department of Rheumatology and Clinical Immunology, Medisch Spectrum Twente, Enschede, The Netherlands

**Keywords:** patient-reported outcomes, central sensitization, pain, content overlap

## Abstract

**Objectives:**

Central pain mechanisms may be prominent in a considerable subset of rheumatology patients with persistent pain. Several self-report instruments have been used in previous research to infer the presence and severity of central sensitization (CS) that stem from different definitions or approaches of CS. The current study aimed to evaluate and quantify the overlap of actual symptoms measured among self-report measures of CS in rheumatology.

**Methods:**

We used Fried’s (2017) comprehensive systematic approach to analyse the content of five commonly used or typical self-report measures (Generalized Pain Questionnaire, Pain Sensitivity Questionnaire, Central Sensitization Inventory, Central Aspects of Pain in the Knee scale and the painDETECT) used in rheumatology research and to visualize and quantify the overlap in symptoms measured.

**Results:**

The five instruments together measured 39 different symptoms, most of which could be grouped into nociplastic pain manifestations (7 symptoms), neuropathic pain qualities (5 symptoms), and psychosomatic symptoms and emotional distress (25 symptoms). Most symptoms (74.4%) were unique to a single instrument. Thermal allodynia was the most frequently measured symptom across the different instruments, assessed in four of the measures. Average content overlap was very low and ranged from no overlap at all to moderate overlap (Jaccard index = 0.43) between pairs of instruments.

**Conclusion:**

There is high heterogeneity and limited overlap in the content of self-report measures used to infer central pain sensitization. This may lead to results that are specific to the particular instrument and may limit the generalizability and comparability of study findings in rheumatology research.

Key messages

- Different self-report instruments are used in rheumatology research to measure central sensitization
- This study showed that the symptoms measured by these instruments vary widely and show limited overlap
- Researchers should be cautious in generalizing their findings or comparing these with studies that used a different instrument

## Introduction

Pain is the prominent symptom of most rheumatic conditions. Traditionally, rheumatic pain has been considered and treated as being nociceptive in nature, resulting from tissue inflammation or damage. However, recent years have seen an increasing interest in the study of mechanisms other than nociceptive pain in patients with different rheumatic conditions. As a result, central pain mechanisms are now widely accepted to also play a key role in a proportion of rheumatology patients presenting with chronic pain [1–4].

Central sensitization (CS) refers to experiences of pain that do not result from obvious signs of damage or inflammation nor neuropathy, but from the altered function of pain-related sensory pathways in the periphery and central nervous system, causing increased sensitivity [5]. This category of pain has been referred to by various names, including centralized pain, chronic widespread pain and (generalized) pain hypersensitivity. The International Association for the Study of Pain (IASP) recently proposed to use the mechanist term nociplastic pain [6, 7].

Central pain mechanisms can be assessed using quantitative sensory testing (QST) or central nervous system imaging techniques such as functional magnetic resonance imaging (fMRI) [8]. As these methods are either complex and time consuming or costly, they are often impractical in both daily clinical care and research settings. Consequently, many studies have relied on self-report measures to measure symptoms of CS or infer the presence of CS in rheumatology patients. However, vastly different questionnaires stemming from different definitions or approaches of CS appear to have been used, which raises the question of generalizability and comparability of the results.

For instance, several studies used the painDETECT [9] questionnaire to assess CS. The painDETECT is a screener for neuropathic pain symptoms based on typical neuropathic pain quality descriptors. As these pain qualities have been shown to correlate with signs of CS as shown with fMRI and QST, the painDETECT has also been suggested as a useful instrument for the identification of pain sensitization [10–12]. Other studies have used the Central Sensitization Inventory (CSI) [13], which is based on a biopsychosocial view of CS. The CSI has been explicitly suggested by some researchers to be a useful measure to detect CS pain [14, 15], although others have criticized its limited concurrent validity with psychophysical tests of CS-related signs and its inability to detect conditioned pain modulation [16, 17]. Some recent studies, on the other hand, used measures specifically focused on a more mechanistic view of CS pain. These measures directly ask about typical sensations indicative of heightened pain sensitivity. For instance, Vriezekolk *et al.* [18] used the Pain Sensitivity Questionnaire (PSQ) [19], while Jansen *et al.* [20] and ten Klooster *et al.* [21] used the Generalized Pain Questionnaire (GPQ) [22]. Finally, the recently introduced Central Aspects of Pain in the Knee (CAP-Knee) questionnaire measures several psychological or somatic effects of pain potentially reflecting central mechanisms [23, 24].

The rationale for using one of these specific self-report instruments in these studies is rarely mentioned and likely based on pragmatic considerations such as familiarity with the instrument or previous studies. Nonetheless, conclusions tend to be drawn about central (pain) sensitization in general, and not about CS as measured by a particular instrument. Given the very different origin and approach of the questionnaires, however, the content of the symptoms assessed by their items also likely differs. If so, the results of CS studies may be specific to the particular instrument used, potentially limiting the generalizability and comparability of study findings [25]. The issue of (lack of) content overlap is not unique to measures of CS, but applicable to many other health-related concepts that are measured by patient-reported outcome measures (PROMs). Fried [25] developed a framework to systematically evaluate and quantify the overlap in symptoms measured by different PROMs that intend or claim to measure the same concept. This approach has now been used for several concepts such as prolonged grief [26] and mental pain [27]. The objective of the current study was to use Fried’s framework to systematically evaluate and quantify the overlap of items among commonly used self-report measures of CS in rheumatology.

## Methods

### Selection of instruments

Selection of the questionnaires for the current study was based on a scan of the literature on CS in combination with the terms rheumatic or arthritis and a recent systematic review of questionnaires to screen for pain sensitization and neuropathic like pain in inflammatory arthritis [28]. We did not aim for exhaustive inclusion of all self-report instruments ever used with the intention of measuring or inferring CS (such as all neuropathic screeners) but rather selected a diverse set of frequently used or typical instruments for illustrative purposes.

The GPQ is a seven-item instrument that assesses the presence and severity of various pain experiences commonly associated with likely generalized pain hypersensitivity. It was purposively developed to cover seven common manifestations of CS based on a literature review and as described by Woolf [5]. Each item is measured on a 5-point Likert-type severity scale [22, 29]. The PSQ was developed as a short self-rating instrument for the assessment of pain sensitivity that provides information similar to experimental pain sensitivity assessments. It consists of 17 items with imagined painful situations that could occur in daily life (e.g. ‘Imagine you burn your tongue on a very hot drink’). Respondents are asked to rate how painful each situation would be for them on a 0–10 NRS [19, 30]. The CSI contains 25 items that measure somatic and emotional complaints often associated with central sensitivity syndromes on a 5-point temporal Likert scale [13, 31]. As such, the CSI was not exclusively intended to measure pain symptoms, but to differentiate between different types of chronic pain patients with different levels of CS impairment [13]. The CAP-Knee questionnaire was recently developed to assess manifestations linked to central mechanisms, including psychological and somatic disturbances, specifically in people with knee pain [24]. The CAP-Knee consists of seven items measuring experiences in the past week using a 4-point Likert scale and an additional manikin for marking the pain area(s). The painDETECT consists of seven items evaluating the experienced severity of specific pain qualities (e.g. burning) commonly associated with neuropathic pain, one evaluating the course pattern of pain, and one evaluating pain radiation. Additionally, the questionnaire contains a manikin for marking the area(s) of pain and three 0–10 numerical rating scales (NRSs) for current, worst, and average pain severity, which are not used in the final score [9, 32]. The painDETECT was never intended for assessing central pain sensitization but has become a frequently used instrument in rheumatology to identify neuropathic pain as a proxy for symptoms related to maladaptive central processing [28].

Together, the instruments contained 66 items that are used for scoring them. The items of all five questionnaires can be found in Supplementary Table S1, available at *Rheumatology Advances in Practice* online.

### Content analysis

Fried developed a comprehensive systematic approach to qualitatively analyse the content of different multi-item self-report measures, intended or used to assess the same construct and to quantify and visualize the overlap in symptoms measured [25, 33]. The first step of this approach consisted of extracting distinct symptoms from the items within each questionnaire. This was done independently by two blinded researchers (J.S. and H.V.) with the instruction to try to stay within three words to describe each symptom measured and to try to stay close to the original wordings used in the items in this stage (e.g. ‘pain from touch’).

The second step consisted of harmonizing the symptom descriptions of both raters and combining similarly worded symptoms between questionnaires to ensure that they were not redundant (e.g. ‘spreading pain’ and ‘radiating pain’). The initial harmonization was performed by PtK and discussed with J.S. and H.V. After consensus was reached on all symptom descriptors, pain-specific symptoms were linked to and labelled according to appropriate pain and CS nomenclature. Pain symptoms and sensations reflecting specific mechanistic pain manifestations were categorized using the latest pain terminology as defined by the IASP [34, 35] and terms used in Woolf’s seminal paper on the mechanisms of CS [5]. For clarity, items measuring a diverse range of physical or emotional symptoms or sensations not directly related to pain mechanisms, which were present only in the CSI and CAPS-Knee, were collapsed into one single category of ‘psychosomatic symptoms and emotional distress’ to reflect the holistic biopsychosocial view and literature on central sensitivity syndromes [36, 37]. Previous studies of the CSI have also suggested that these symptoms all tend to tap into a single general underlying domain of CS [38, 39].

### Statistical analysis

Statistical analysis and visualizations were performed by adapting the R code provided by Fried [40]. To visualize the content overlap, the different symptoms in the instruments were depicted in a circular plot using the ggplot2 package. Next, the content overlap was quantitatively estimated using the Jaccard index. The Jaccard index is a similarity coefficient for binary data that ranges from 0 (no overlap among scales) to 1 (complete overlap). In this case, the index is calculated as the number of symptoms that two instruments share divided by the total number of unique symptoms in both instruments. In accordance with Fried [25], Jaccard indices between two instruments were considered very weak (0.00–0.19), weak (0.20–0.39), moderate (0.40–0.59), strong (0.60–0.79) or very strong (0.80–1).

### Ethics

No humans or animals were involved in this study, therefore ethics approval was not required.

## Results

**Figure 1.**
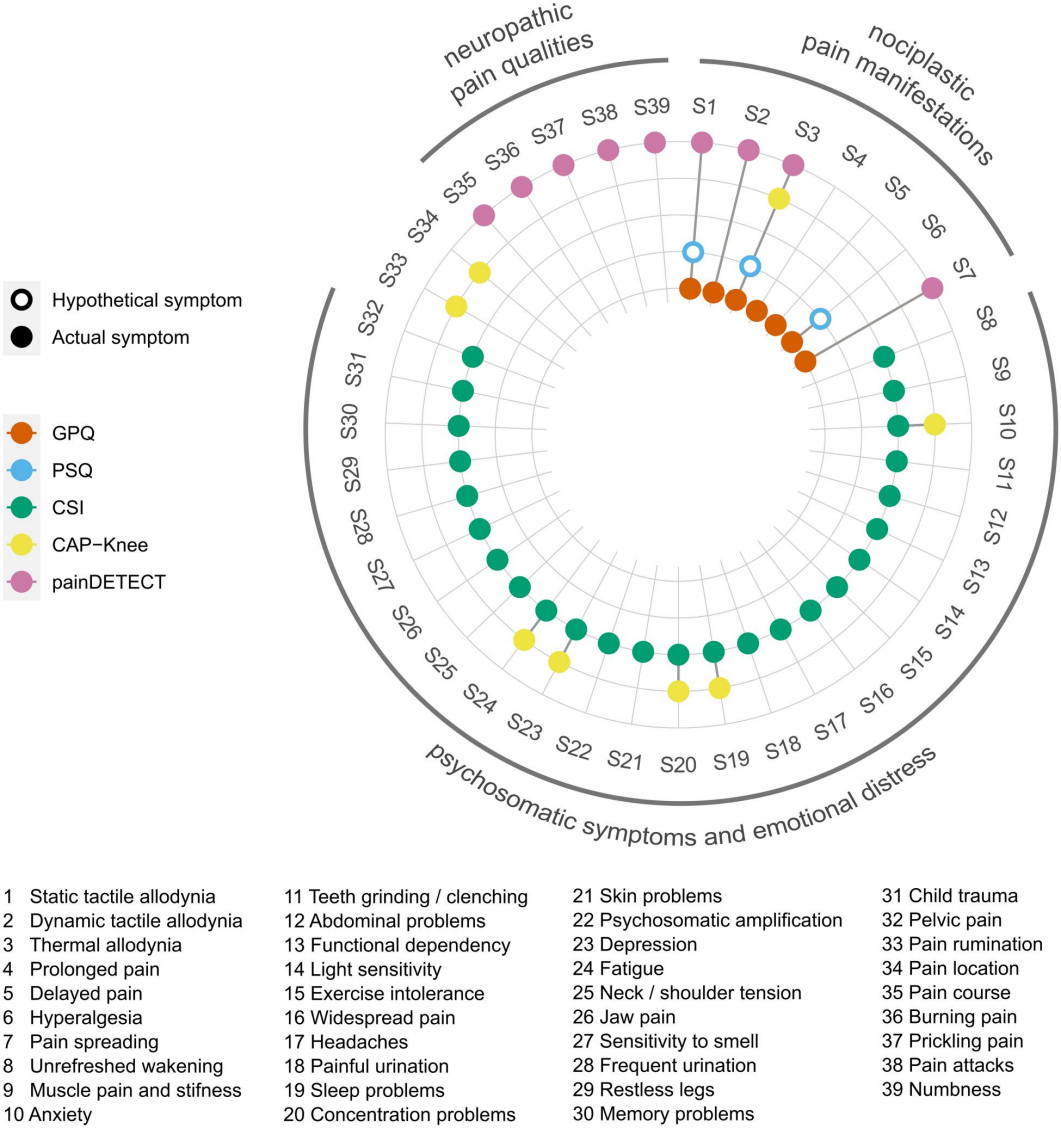
Co-occurrence of 39 central sensitizsation symptoms across the five self-report instruments. GPQ: Generalized Pain Questionnaire [22]; PSQ: Pain Sensitivity Questionnaire [19]; CSI: Central Sensitization Inventory [13]; CAP-Knee: Central Aspects of Pain in the Knee scale [24]; painDETECT: painDETECT questionnaire [9]

The content analysis of the 66 items resulted in 39 unique symptoms measured across the five different instruments (see Fig. 1). All items of the GPQ, CSI, CAP-Knee and painDETECT measured a single unique symptom within their respective instrument. Although the PSQ has 17 items, 14 of its items measure different examples of possible hyperalgesia, while examples of thermal and static tactile allodynia are measured by 2 and 1 items, respectively. The PSQ is also the only instrument that asks subjects to estimate their symptoms across a variety of hypothetical daily activities or situations, instead of actual experienced symptoms. The vast majority of measured symptoms across the instruments (37/39; 94.9%) could be further grouped into three overarching categories: mechanistic nociplastic pain manifestations (7 symptoms), neuropathic pain qualities (5 symptoms) and psychosomatic symptoms and emotional distress (25 symptoms). The CAP-Knee additionally contains one item related to coping (pain rumination) and one mannikin item assessing the pain location(s).

On average, the 39 different symptoms appear in only a mean of 1.33 (median = 1, mode = 1) of the questionnaires. Of the 39 symptoms, 29 (74.4%) were unique to a single instrument. Thermal allodynia was the most frequently measured symptom across the different instruments and was assessed in all measures except the CSI.

Figure 1 shows how the different approaches to CS underlying the instruments is reflected in different symptom contents. Both the GPQ and the PSQ only measured nociplastic pain manifesting. However, the GPQ assessed additional typical manifestations other than hyperalgesia and allodynia, such as prolonged pain and pain spreading. The CSI measures only psychosomatic symptoms and emotional distress and no specific nociplastic or neuropathic pain symptoms. The painDETECT measures a mix of neuropathic pain symptoms (pain quality descriptors) and nociplastic pain manifestations (different types of allodynia). The recently developed CAP-Knee has the most mixed content and measures psychosomatic symptoms and emotional distress (five items), thermal allodynia (one item) and two symptoms that don’t seem to match with any of the main overarching approaches (categories).

The limited overlap in content between the different instruments was confirmed by their Jaccard similarity indices (Table 1). The mean overlap between all five instruments is only 0.11 (median = 0.12), which indicates a very weak content similarity. Only the GPQ and PSQ, which are both based on a mechanistic on CS pain, show moderate content overlap (*J* = 0.43). Both the GPQ (*J* = 0.33) and the PSQ (*J* = 0.20) weakly overlap with the painDETECT. The CSI and CAP-Knee have very weak or no overlap at all with the other three instruments.

**Table 1.** Overlap in sensitisation symptoms of the 5 self-report instruments

	GPQ	PSQ	CSI	CAP-Knee	painDETECT
GPQ	1				
PSQ	0.43	1			
CSI	0.00	0.00	1		
CAP-Knee	0.07	0.10	0.18	1	
painDETECT	0.33	0.20	0.00	0.06	1
Mean overlap	0.17	0.15	0.04	0.08	0.12

Values are Jaccard similarity coefficients which can range from 0 (no overlap) to 1 (total overlap).

GPQ: Generalized Pain Questionnaire; PSQ: Pain Sensitivity Questionnaire; CSI: Central Sensitization Inventory; CAP-Knee: Central Aspects of Pain in the Knee.

## Discussion

The aim of the current study was to systematically evaluate the overlap in symptoms addressed in several self-report measures that have all been used to infer CS in rheumatology populations. Using Fried’s comprehensive stepwise approach [25], the findings showed high heterogeneity and low overlap in the content of the instruments. Different instruments assessed either mechanistic nociplastic pain manifestations, psychosomatic symptoms and emotional distress, neuropathic pain qualities, or combinations of these symptom categories. Although the different instruments have all undergone thorough psychometric testing in the past, the considerable differences in their content suggest that study findings based on these measures may not be generalizable and comparable. Future researchers are suggested to explicitly state their definition or approach of central (pain) sensitization and select an instrument which content best fits this approach.

CS is a complex and very broadly utilized concept, which development and experienced manifestations can be viewed and approached from both a more biomedical mechanistic or a more biopsychosocial point of view [5, 36, 41, 42]. Much research has specifically focused on the pathophysiological mechanisms and manifestations of CS. As a result, the identification of CS is often aimed at identifying altered pain perceptions such as neuropathic-like or centrally-mediated pain symptoms [41]. Other researchers have convincingly argued that CS is not a purely biological phenomenon and that patients with CS also often present with anxiety, stress and depression and symptoms like endocrine dysfunction, psychosocial distress, trauma and disrupted sleep [36]. Therefore, they argue that CS represents a biopsychosocial model, requiring the evaluation of these symptoms as well.

The current study shows that these different approaches are also reflected in the content of the different self-report measures used to infer CS. The painDETECT contains typical neuropathic pain descriptors, which have been shown to correlate with signs of CS, and also measures different types of allodynia which are also typical for centrally mediated pain. The PSQ and GPQ both contain only items directly targeting pain (hyper-)sensitivity symptoms, with the GPQ measuring a more complete array of typical CS pain features according to Woolf [5]. The CSI, on the other hand, measures a wide array of psychological and physical symptoms frequently seen in patients with widespread pain or fibromyalgia, as also described in the biopsychosocial model of CS [36]. Interestingly, the CSI actually does not measure a single pain manifestation typical for centralized pain mechanisms (either neuropathic-like or nociplastic). Finally, the recently developed CAP-Knee scale contains a mix of psychological and psychosomatic symptoms, coping and pain characteristics.

All five self-report measures evaluated in the current study have shown good internal psychometric properties in the past, and all may arguably be valuable for assessing CS in rheumatology patients. However, the (very) low overlap in actual symptoms they measure suggests that they cannot be used interchangeably to measure or infer CS. It is unclear to what extent this lack of or limited overlap in symptoms measured actually poses a threat to the generalizability and comparability of research on CS in rheumatology research in practice. Previous studies do suggest that the different self-report measures are strongly intercorrelated in different patient populations. For instance, although there is no overlap in symptoms measured by the GPQ and CSI, their total scores have previously been shown to correlate strongly (*r* = 0.70) in RA patients [20]. And despite their weak overlap in item content, the GPQ and painDETECT even correlated at 0.87 in RA and FM patients [22]. Such strong correlations could be taken to suggest that, even though they encompass different symptoms, they essentially measures the same underlying CS concept at the total score level. However, high intercorrelations do not necessarily mean that they can also be used interchangeably or that different measures lead to generalizable and comparable results. Several studies have shown that CSI scores were not or only weakly correlated with QST of pain sensitivity [17, 43], while the PSQ and GPQ have shown moderate to strong correlations with several QST measurements such as pressure pain and heat pain thresholds [20, 30]. Moreover, the CSI and PSQ have been shown to correlate very differently with measures of emotional distress (depression, anxiety, stress, negative affect), with observed correlations ranging between 0.64 and 0.67 for the CSI and between 0.14 and 0.31 for the PSQ [44]. It was also found that the PSQ, but not the CSI, was weakly associated with QST measurements but that neither was clearly associated with a pattern of widespread pain sensitivity.

Given these findings and the observed limited content overlap of self-report measures that have been used in rheumatology research, it is very well possible that the results of these studies are specific to the particular instrument—and thus approach of CS—used. This would limit the generalizability and comparability of study findings in rheumatology. Therefore, future studies are encouraged to clearly define the approach of CS used in their study and to select a self-report measure that best matches with this approach. The content analysis results of the current study can aid researchers in this decision. For studies that take a biopsychosocial approach of CS, the CSI may be a good option. For those that are more interested in assessing the actual pain manifestations that are typical for CS, the PSQ or GPQ may be better suited. Although the painDETECT has been quite frequently used as a surrogate measures of centralized pain, and has been shown to correlate with pain sensitization, it is a measure of neuropathic pain and rheumatic patients generally have no apparent lesions of the nervous system. Consequently, researchers may instead opt for the PSQ to measure hyperalgesia and allodynia or the GPQ to measure a wider array of typical pain sensitivity symptoms. The current study provides a first step in comparing self-reported instruments to measure central (pain) sensitization in rheumatology studies and suggests that attention should be paid to the different contents assessed in these instruments when selecting an instrument or interpreting study findings. Future empirical studies and reviews are needed that look beyond item content overlap only and that focus on systematic head-to-head comparisons of the actual psychometric performance of the different (total) scale scores of the instruments in terms of construct validity and criterion validity. Together, such findings are likely to allow researchers to make better justified decisions for selecting the most appropriate self-report measure of CS for their study purposes.

This is the first study that systematically evaluated and compared the content of different self-report instruments that are being used to measure centralized pain in rheumatology studies. It should be noted that we did not aim to identify all self-report instruments that have ever been used to infer centralized pain in patients with rheumatic diseases. Instead, a pragmatic selection of the most commonly or typical instruments was made based on one previous systematic review [28] and a scan of the literature for illustrative purposes. To the best of our knowledge, the five instruments selected for the current study make up for the vast majority of studies in rheumatology that have measured central (pain) sensitization by means of a standardized self-report instruments and cover the different types of instruments used. There are, however, several studies that used neuropathic pain screeners other than, but very similar to the content of, the painDETECT such as the Douleur Neuropathique 4 (DN4) scale. Nonetheless, it is not expected that including all self-report measures would have considerably changed the current observation of limited content overlap in the instruments.

In conclusion, currently used self-report measures of CS in rheumatology show high heterogeneity and remarkably limited overlap in the symptoms they measure. Future studies are suggested to clearly define their approach of CS, select instruments that match with this approach, and to be cautious in generalizing their findings or comparing these with studies that used a different instrument.

## Supplementary Material

rkae108_Supplementary_Data

## Data Availability

All data and software code underlying this article are available on GitHub, at https://github.com/kloosterpm/content_overlap_cs
