# Supplementary material for: Limited content overlap between commonly used self-report instruments for central (pain) sensitization in rheumatology
Source: Rheumatol Adv Pract. 2024 Aug 26;8(3):rkae108. doi: 10.1093/rap/rkae108 (PMC11374029; doi:10.1093/rap/rkae108)
Supplement: rkae108_Supplementary_Data [file rkae108_supplementary_data.pdf]

Supplementary Table S1: Item content of the five questionnaires

Questionnaire	Item
Generalized Pain Questionnaire (GPQ)	1. Pain from light touch (eg, from a pat on the back or handshake)
	2. Pain from friction on skin (eg, from clothing or the wind)
	3. Pain from heat or cold that most people would not experience as painful (eg, from cold water or holding cold objects)
	4. Pain that lasts longer than with most other people
	5. Pain that arises only later and that wouldn't arise in most other people (eg, hours later or the next day after exertion, such as walking)
	6. Unusually intense experiences of pain (eg, nausea or gasping for air)
	7. Pain that also spreads to other parts of the body (eg, pain in the hand that spreads to the underarm when holding objects)
Pain Sensitivity Questionnaire (PSQ)	1. Imagine you bump your shin badly on a hard edge, for example, on the edge of a glass coffee table. **How painful would that be for you?**
	2. Imagine you burn your tongue on a very hot drink.
	3. Imagine your muscles are slightly sore as the result of physical activity.
	4. Imagine you trap your finger in a drawer.
	5. Imagine you take a shower with lukewarm water.
	6. Imagine you have mild sunburn on your shoulders.
	7. Imagine you grazed your knee falling off your bicycle.
	8. Imagine you accidentally bite your tongue or cheek badly while eating.
	9. Imagine walking across a cool tiled floor with bare feet.
	10. Imagine you have a minor cut on your finger and inadvertently get lemon juice in the wound.
	11. Imagine you prick your fingertip on the thorn of a rose.
	12. Imagine you stick your bare hands in the snow for a couple of minutes or bring your hands in contact with snow for some time, for example, while making snowballs.
	13. Imagine you shake hands with someone who has a normal grip.
	14. Imagine you shake hands with someone who has a very strong grip.
	15. Imagine you pick up a hot pot by inadvertently grabbing its equally hot handles.
	16. Imagine you are wearing sandals and someone with heavy boots steps on your foot.
	17. Imagine you bump your elbow on the edge of a table ("funny bone").
Central Sensitization Inventory	1. I feel tired and unrefreshed when I wake from sleeping.
	2. My muscles feel stiff and achy.
	3. I have anxiety attacks.
	4. I grind or clench my teeth.
	5. I have problems with diarrhea and/or constipation.
	6. I need help in performing my daily activities.
	7. I am sensitive to bright lights.
	8. I get tired very easily when I am physically active.
	9. I feel pain all over my body
	10. I have headaches.
	11. I feel discomfort in my bladder and/or burning when I urinate.
	12. I do not sleep well.
	13. I have difficulty concentrating.
	14. I have skin problems such as dryness, itchiness, or rashes.
	15. Stress makes my physical symptoms get worse.
	16. I feel sad or depressed.
	17. I have low energy.
	18. I have muscle tension in my neck and shoulders.
	19. I have pain in my jaw.
	20. Certain smells, such as perfumes, make me feel dizzy and nauseated.
	21. I have to urinate frequently.
	22. My legs feel uncomfortable and restless when I am trying to go to sleep at night.
	23. I have difficulty remembering things.
	24. I suffered trauma as a child.
	25. I have pain in my pelvic area.
Central Aspects of Pain in the Knee Scale	1. Cold or heat (e.g., bath water) on my knee was painful
	2. I generally felt tired

	3. My knee pain stopped me concentrating on what I was.
	4. I kept thinking about how much my knee hurts.
	5. In general, I got sudden feelings of panic.
	6. My knee pain affected my sleep.
	7. I generally still enjoyed the things I used to enjoy.
	8. This final question is about pain you may have had in any part of your body. Please shade in the diagram below, to indicate where you have suffered any pain for most days in the last four WEEKS. By pain we also mean aching and/or discomfort. Please do not include pain due to feverish illness such as flu.
painDETECT	Mark the picture that best describes your pain:
	Do you suffer from a burning sensation (e.g. stinging nettles) in the marked area?
	Do you have a tingling or prickling sensation in the area of your pain (like pins and needles or electrical tingling)?
	Is light touching (clothing, a blanket) in this area painful?
	Do you have sudden pain attacks in the area of your pain, like electric shocks?
	Is cold or heat (e.g. bath water) in this area occasionally painful?
	Do you suffer from a sensation of numbness in the area that you marked?
	Does slight pressure in this area, e.g. with a finger, trigger pain?
	Does your pain spread to other regions of your body?
	Please mark your main area of pain
